# Design and Application of a High Sensitivity Piezoresistive Pressure Sensor for Low Pressure Conditions

**DOI:** 10.3390/s150922692

**Published:** 2015-09-08

**Authors:** Huiyang Yu, Jianqiu Huang

**Affiliations:** 1 College of Computer Science and Technology, Nanjing Tech University, No.30, South Puzhu Road, Pukou District, Nanjing, 211800, China; 2 Key Laboratory of MEMS of the Ministry of Education, Southeast University, No.2 Sipailou, Xuanwu District, Nanjing 210096, China; E-Mail: hjq@seu.edu.cn

**Keywords:** piezoresistive, pressure sensor, stress concentration, low pressure

## Abstract

In this paper, a pressure sensor for low pressure detection (0.5 kPa–40 kPa) is proposed. In one structure (No. 1), the silicon membrane is partly etched to form a crossed beam on its top for stress concentration. An aluminum layer is also deposited as part of the beam. Four piezoresistors are fabricated. Two are located at the two ends of the beam. The other two are located at the membrane periphery. Four piezoresistors connect into a Wheatstone bridge. To demonstrate the stress concentrate effect of this structure, two other structures were designed and fabricated. One is a flat membrane structure (No. 2), the other is a structure with the aluminum beam, but without etched silicon (No. 3). The measurement results of these three structures show that the No.1 structure has the highest sensitivity, which is about 3.8 times that of the No. 2 structure and 2.7 times that of the No. 3 structure. They also show that the residual stress in the beam has some backside effect on the sensor performance.

## 1. Introduction

Nowadays, energy and environmental problems are of great concern and pressure sensors play an important role in environmental monitoring [1]. They are widely used in weather stations [2,3]. Additionally, with the development of the Internet of Things (IOT), their application area has been greatly expanded, which also brings some special requirements to sensor performance. In some instruments, low pressure detection is required and this demands higher sensor sensitivity to get enough resolution. For example, a radiosonde carries along several sensors, including a pressure sensor. The pressure sensor is used to detect the pressure in the upper air, where the pressure is much lower than on the Earth’s surface. Hence, the sensitivity of the pressure sensor must be improved to perceive small pressure changes. Another situation where low pressure sensors can be used is height detection. With a high sensitivity low pressure sensor, the altitude variation of even one stair can be sensed. Accordingly, the usage of low pressure sensors can be expended to spatial orientation and 3-D navigation. There have been a few reports on low pressure sensors. Li *et al*. reported a SOI Pirani sensor that can test the pressure ranging from 10 pa to 26.6 kPa, but it has high power consumption and is difficult to fabricate [4]. In early 1990s, Bao *et al*. reported for the first time beam-membrane structures for stress concentration to improve the sensitivity of pressure sensors [5,6,7]. In 2010, Tian *et al*. were the first to report a cross-beam structure [8]; the sensor is proved to show improved sensitivity under low pressure conditions. Their research also showed that stress has a great relation with beam thickness. In their designation, the dimension of the membrane is 2900 μm × 2900 μm × 20 μm and the beam thickness is about 30 μm. This size is too large for our application. When sensor dimensions especially the membrane thickness, are scaled, the nonlinearity between applied pressure and stress must be considered. Hence, when the total thickness of the beam and the membrane is settled, there must be a compromise between them. In this paper, an enhanced cross-beam membrane structure is designed and provides a way to solve this problem. Additionally, the sensor in this paper is fabricated with a CMOS compatible process.

Since the emergence of Micro-electro-mechanical systems (MEMS) many kinds of MEMS pressure sensors have been reported. According to their sensing principles, they can be divided into piezoresistive [9], capacitive [10], resonant [11] and so on. Among them, the piezoresistive pressure sensors are the most used because of their significant advantages such as high sensitivity, excellent linearity and repeatability [12]. Their principle is that the resistance of a piezoresistor changes when the piezoresistor is exposed to stress. The resistance of a cuboid resistor can be expressed by the following Equation [8,13,14,15,16]:

(1) $$R=\rho \frac{l}{A}$$

Here, *l*, *ρ*, *A* are length, resistivity and cross sectional area of the resistor. When expressed as a differential equation, the above equation becomes: 

(2) $$\frac{△R}{R}=\frac{△l}{l}-\frac{△A}{A}+\frac{△\rho }{\rho }$$

From the two equations above, it can be concluded that three factors are related to the resistance: the resistor length, the cross sectional area as well as resistivity of the resistor. The former two factors are called the dimensional effect. The last one is called the physical effect. For a piezoresistor exposed to stress, the dimensional effect is usually small and can be neglected. Hence, Equation (2) can be written as follows:

(3) $$\frac{△R}{R}=\frac{△\rho }{\rho }=\pi_{l}\sigma_{l}+\pi_{t}\sigma_{t}$$

Here, *π_l_*, *π_t_* are the longitudinal and the transversal piezoresistive coefficients, the values of which are determined by material properties of piezoresistors. For silicon, these values are also related to silicon crystal orientation. σ*_l_*, σ*_t_* are the longitudinal and the transversal stresses, which are determined by the load or pressure applied on the piezoresistor. For a flat membrane, when the membrane suffers pressure and deflects, the maximum longitudinal stress generated around the middle of the membrane edge. Accordingly, the piezoresistors are usually laid around this area [17]. Under small deflection conditions, the maximum stress can be expressed as [18,19,20,21,22]:

(4) $${\left(\sigma_{l}\right)}_{\max}=\frac{3a^{2}p}{2\pi^{2}h^{2}}$$

(5) $${\left(\sigma_{t}\right)}_{\max}=\frac{3va^{2}p}{2\pi^{2}h^{2}}$$

In the equations above, *a*, *h*, *p* are the half length of membrane diameter, thickness of membrane and pressure applied on the membrane, respectively. The maximum stress is proportional to the applied pressure and inversely proportional to the square of the membrane thickness. From these two equations, it can be seen that reducing the membrane thickness is an efficient way to increase the stress. However, the membrane thickness cannot decrease infinitely, because it will lead to nonlinear effects between stress and applied pressure. This nonlinear effect is undesirable for an ideal sensor. On this account, beam-membrane structures are developed. When a beam-membrane deforms, the stress will concentrate in the beam.

## 2. Experimental Section 

For a beam-membrane structure, the equivalent membrane thickness can be calculated by the following Equation [23]:

(6) $$h_{eq}=\frac{A}{2a}$$

Here, *A* is the cross section area of the beam-membrane. As we know, the maximum stress in a membrane is usually inversely proportional to the membrane thickness. Hence, for a beam-membrane structure with settled total thickness, reducing the membrane thickness, which reduces the cross section area, is beneficial for stress concentration. This conclusion has also been proved in [8]. However, the membrane thickness must be large enough to avoid nonlinear effects. Hence, there must be a compromise between beam and membrane thickness. In our design, the total thickness is 13 μm and the membrane diameter is 900 μm. The allowed beam thickness is very limited. Here, a method is figured out to solve this problem. An extra aluminum beam is fabricated above the silicon beam to increase the total thickness of the beam.

**Figure 1:**
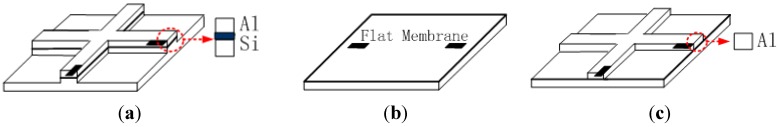
Three structures designed to demonstrate the stress concentration effect: (**a**) The structure with a crossed silicon beam and aluminum beam (No. 1); (**b**) The flat membrane structure (No. 2); (**c**) The structure with aluminum beam but no silicon beam (No. 3).

The Number 1 sensor reported in this paper (No. 1 in Figure 1a) is developed on the basis of ordinary piezoresistive pressure sensors. In an ordinary pressure sensor, there are four pizeoresistors fabricated in a flat membrane. Two of them are located vertical to the membrane side. Their resistances increase as pressure is applied on the membrane. The other two are parallel to the membrane side and their resistances decrease as pressure is applied. These piezoresistors connect into a Wheatstone bridge. The sensor designed here has four piezoresistors, a square membrane and a crossed beam on the membrane. The beam, used for stress concentration, consists of three layers as shown in Figure 1a. They are the silicon layer, aluminum layer and isolation layer. Four piezoresistors are buried in the surface of the silicon layer. Two piezoresistors are on the beam, the resistances of which increase as pressure is applied, another two piezoresistors are in the area outside the membrane region. The resistances of these two piezoresistors remain unchanged when pressure changes. These four piezoresistors connect into a Wheatstone bridge. Both the silicon beam and the aluminum beam contribute to the stress acting on the piezoresistors. To prove this conclusion, another two structures are fabricated, one is the flat membrane structure Number 2 (No. 2) (shown in Figure 1b). It has no beam on the membrane. The other is also a cross-beam structure (Number 3, No. 3) (illustrated in Figure 1c). The beam in this structure only has an aluminum layer. That is to say, an aluminum beam is attached on a flat membrane.

**Table 1:** The material parameters used for the FEM simulation.

Parameters	Value
Thickness of silicon (μm)	13
Thickness of LPCVD SiO_2_ (μm)	0.3
Thickness of LPCVD Si_3_N_4_ (μm)	0.1
Thickness of Aluminum (μm)	2
The Young’s Modulus of silicon (GPa)	130
The Young’s Modulus of SiO_2_ (GPa)	73
The Young’s Modulus of Si_3_N_4_ (GPa)	254
The Young’s Modulus of Aluminum(GPa)	77
The Poisson of silicon	0.25
The Poisson of SiO_2_	0.17
The Poisson of Si_3_N_4_	0.28
The Poisson of Aluminum	0.33

For a beam-membrane structure, the above Equations (4) and (5) for stress calculation are not suitable any more. FEM simulations are used to analyze the stress distribution in the membrane and decide the width and thickness of the beam. The material parameters used in simulations are listed in Table 1. 

### 2.1. The Beam-Thickness and the Stress

**Figure 2:**
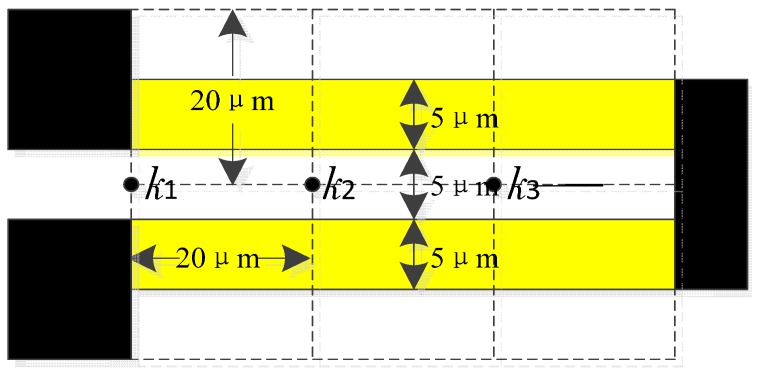
The dimensions of the piezoresistors and the mesh element distribution in the simulation.

In this simulation, four dimensions of beam-thickness are chosen, which are 2, 5, 7 and 9.5 μm, respectively. A 2 μm beam-thickness means that the silicon membrane is not etched and corresponds to the No. 3 structure. A 9.5 μm beam-thickness means that the silicon membrane is etched 7.5 μm deep. According to the membrane deformation simulations, this is the maximum depth allowed, or else the membrane will touch the substrate at 40 kPa. In this simulation, it is assumed that the beam-width is 35 μm, the minimum size decided by the piezoresistor dimensions and fabrication conditions. The dimensions of the piezoresistors and the mesh element distribution are shown in Figure 2.

**Figure 3:**
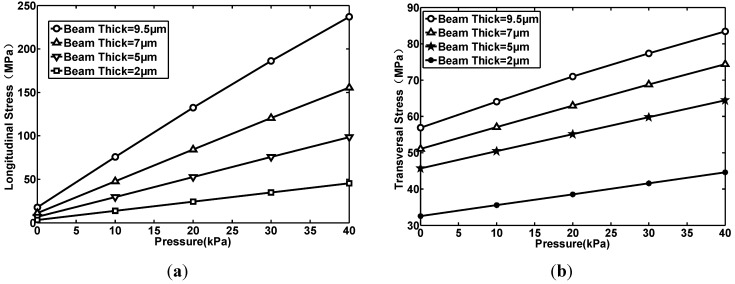
The relationship between pressure and the average stress acting on piezoresstors when beam thick is different: (**a**) The longitudinal stress; (**b**) The transversal stress.

Here, the mean value of stresses in node *k*_1_, *k*_2_ and *k*_3_ are used to represent the average value acting on the whole piezoresitor. Both the average longitudinal stress (shown in Figure 3a) and the transversal stress (shown in Figure 3b) acting on a piezoresistor are extracted. 

By simulation, the relations between beam thickness and the stress can be summarized as follows: (1) The stress is proportional to beam thickness; (2) The beam thickness has a greater effect on longitudinal stress than on transversal stress.

### 2.2. The Beam-Width and the Stress

**Figure 4:**
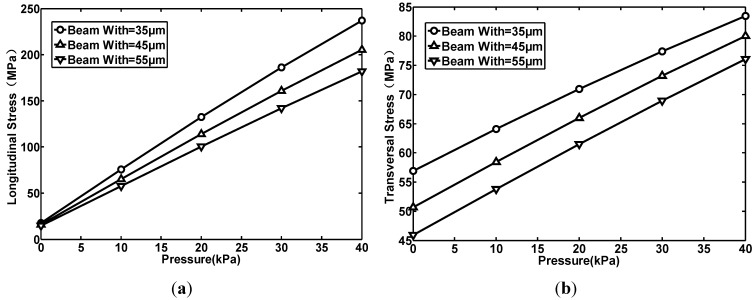
The relationship between pressure and the average stress acting on piezoresstors when the beam width is different: (**a**) The longitudinal stress; (**b**) The transversal stress.

In this simulation, the beam thickness is set as 9.5 μm. Three kinds of beam-width are chosen: 35, 45 and 55 μm. Both the average longitudinal stress (shown in Figure 4a) and the transversal stress (shown in Figure 4b) acting on a piezoresistor are extracted. By simulation, the relation between beam-width and the stress can be summirized as follows: (1) The stress is inversely proportional to beam width; (2) The beam width has a greater effect on the longitudinal stress than on the transversal stress; (3) The beam-width has less of an effect on stress than the beam thickness. In the No. 1 structure, the beam is designed to be 9.5 μm thick and 35 μm wide.

### 2.3. The Residual Stress and the Stress

**Figure 5:**
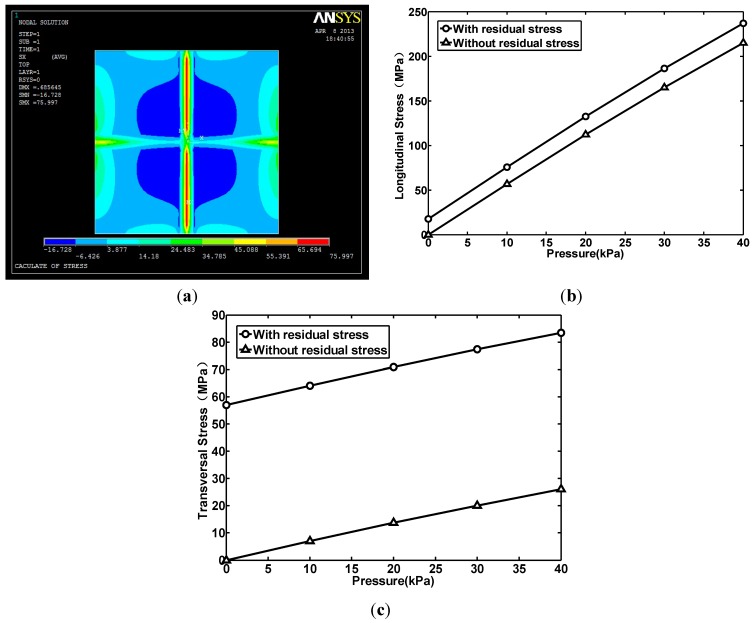
The relationship between pressure and average stress acting on piezoresistors when residual stresses are considered and not considered: (**a**) The stress distribution in the membrane in the x-axis direction under a pressure of 10 kPa with residual stress concerned; (**b**) The longitudinal stress; (**c**) The transversal stress.

It was found after fabrication that the beam-membrane had residual stress and this induced some effects on the testing results. The residual stress has three sources: the LPCVD SiO_2_ and Si_3_N_4_ and the deposited aluminum. The LPCVD SiO_2_ and Si_3_N_4_ are the isolation layers between the aluminum and silicon. The SiO_2_ and Si_3_N_4_ are 0.3 μm and 0.1 μm thick, respectively. Figure 5 shows the simulation results when the residual stress in every material of the beam is taken into consideration. According to process parameters provided by our fabrication foundry, the residual stress in the LPCVD SiO_2_ layer is about −200 MPa (Megapascals). In LPCVD Si_3_N_4_, the residual stress is about 1 GPa (Gigapascals). In deposited aluminum, the residual stress is about −100 MPa. In comparison, the simulation results without considered residual stress are also given. The simulation results prove that the residual stress had some backside effect on the sensor. Firstly, it causes a stress offset when no pressure is applied. Secondly, it reduces the longitudinal stress and enlarges the transversal stress. The transversal stress is even larger than the longitudinal stress when the pressure is low to a certain extent. This causes the output voltage of the sensor to become negative.

**Figure 6:**
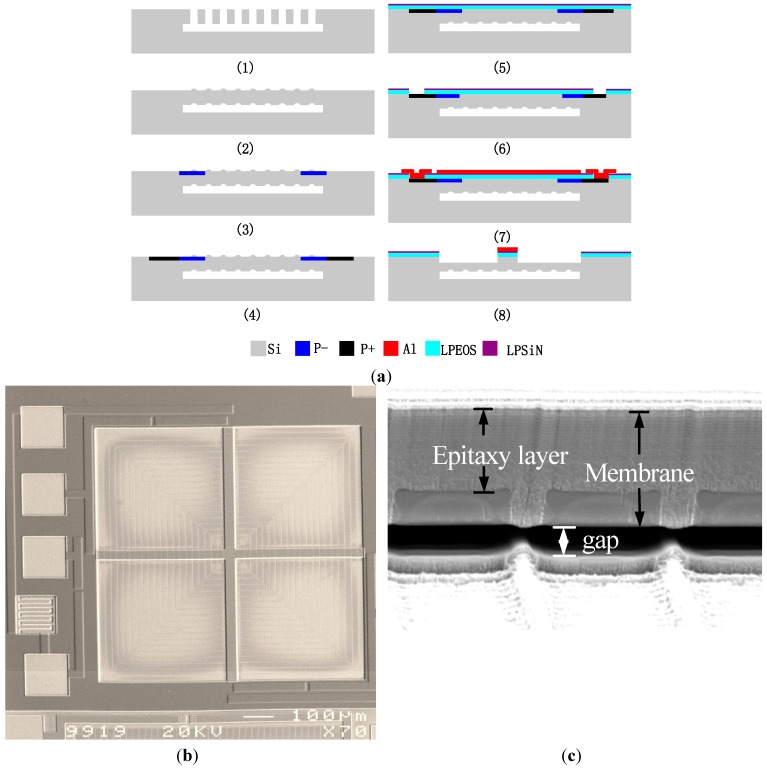
The fabrication process and the SEM photographs of the No. 1 sensor: (**a**) The sensor fabrication process; (**b**) A top view of the sensor; (**c**) A side view of the sensor membrane.

The fabrication process of the sensor, which is compatible with CMOS process, is shown in Figure 6a: (1) Anisotropic dry-etching of the silicon is used to generate some silicon grooves and then the silicon is isotropically dry-etched to release the membrane architecture; (2) The grooves are filled with silicon by an epitaxy process. Consequently, an intact silicon membrane and a sealed cavity are formed. The dimensions of the membrane are 900 μm × 900 μm × 13 μm. The cavity is 5 μm high. The pressure in the cavity is very small and approximates vacuum; (3) Boron ions are implanted on part of the silicon membrane to form the piezoresistors; (4) A higher amount of boron ions is implanted again to form a heavily doped area for ohmic contact; (5) SiO_2_ and Si_3_N_4_ films are deposited by LPCVD to protect the sensor surface and also be the electrical insulation; (6) The deposited SiO_2_ and Si_3_N_4_ are lithographed and etched to expose the contacting pad; (7) 2.0 μm thick aluminum is deposited and patterned to realize electrical connections and form the aluminum beam; (8) The anisotropic dry-etching of the silicon is carried out to etch part of the membrane and form the silicon beam. The etched silicon thickness is about 7.5 μm. For the No. 2 and No. 3 structures, step (8) must be neglected. In this step, the aluminum beam also works as the etching mask of silicon beam. SEM pictures of fabricated sensor are shown in Figure 6b,c. In Figure 6b, the membrane, the cross beam as well as the contact pads can be seen. 

## 3. Results and Discussion

**Figure 7:**
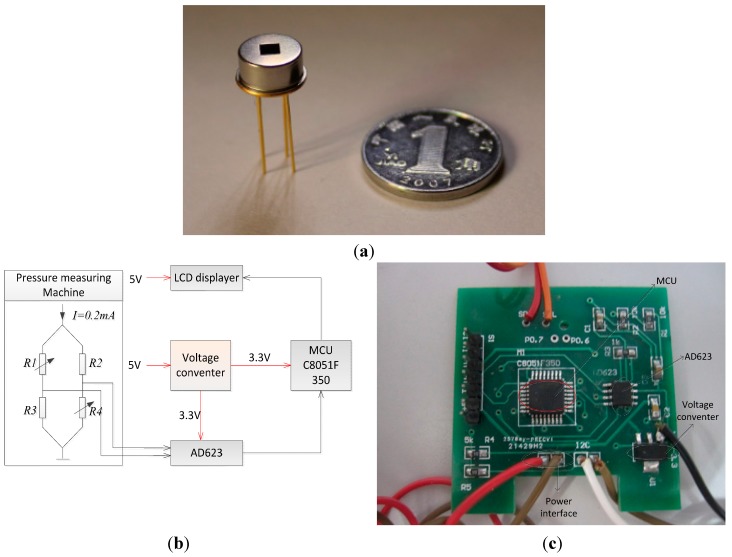
The packaged sensor and the measurement system: (**a**) Picture of the packaged sensor; (**b**) Measurement system of the sensor; (**c**) Photograph of the measurement system circuit.

After three kinds of sensors are diced and wire-bonded, the measurements are carried out. Figure 7a is a picture of a packaged sensor. The diagrams and picture of the measurement system is shown in Figure 7b,c. The pressure controlling machine produces the needed low pressure conditions ranging from 0.5 kPa to 40 kPa. To make the measurements easier and get rid of noise, a signal processing circuit is designed. The output voltage of the sensors are firstly connected to an AD623 amplifier and amplified 100 times. With the help of the Analog/Digital (A/D) converter of a C8051F350 microcontroller unit the amplified analog voltage is converted to a digital signal and then displayed by a LCD display.

**Figure 8:**
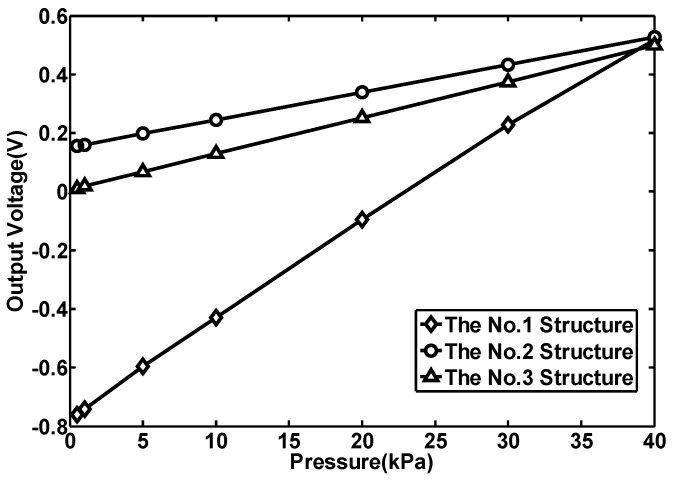
The sensitivity measurement results of the No.1, No.2 and No.3 structures.

The sensitivity measurement results are shown in Figure 8, where the y-axis corresponds to the voltage displayed by the LCD display and the x-axis corresponds to the pressure applied on the membrane. From the results, it can be seen that the output voltages of the three sensors all increase with the applied pressure. For the No. 1, No. 2 and No. 3 structures, the sensitivity is 329 μV/kPa, 89.9 μV/kPa and 124 μV/kPa, respectively. The linearity error of these three structures is 1.69%, 0.23% and 0.38%, respectively. The linearity error of the No. 2 and No. 3 structures is small. The No. 1 structure has the highest linearity error, which is mainly caused by the thinner membrane, but it is within allowance. The No. 1 structure has the highest sensitivity, which is about 3.8 times that of the No. 2 structure and 2.7 times that of the No. 3 structure. Comparing the sensitivity of the No. 1 and the No. 3 structures with the No. 2 structure, it is seen that the cross-beam membrane is able to improve the sensitivity of the sensor and the stress concentration effect has much to do with the beam thickness. It also indicates that both the silicon layer and the aluminum layer of the beam contribute to the sensitivity of the sensors. Besides that, the residual stress, mainly existing in the materials of the beam, has some effect on the output voltage. This effect becomes obvious as the pressure gets low and the output voltage becomes a negative value when the pressure gets below 20 kPa. This situation has been simulated with ANSYS software as shown in Figure 5. As the pressure decreases, the longitudinal stress gets smaller than the transversal stress. This makes the resistance of the piezoresistors on the membrane decrease under pressure. That is why the output voltage becomes a negative value when the pressure is below 20 kPa.

**Figure 9:**
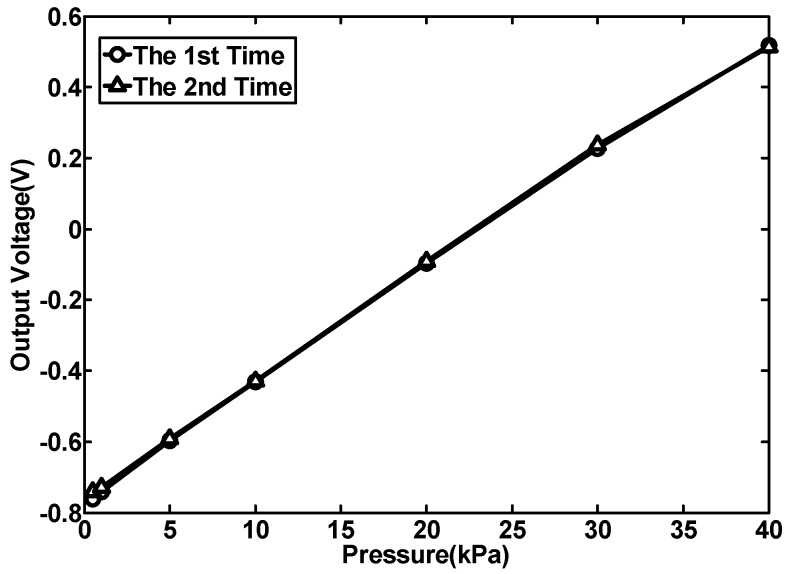
Repeatability measurement results of the No. 1 structure.

To study the repeatability of the sensor, multiple measurements were carried out. Figure 9 gives the test results in two measurements. 

**Figure 10:**
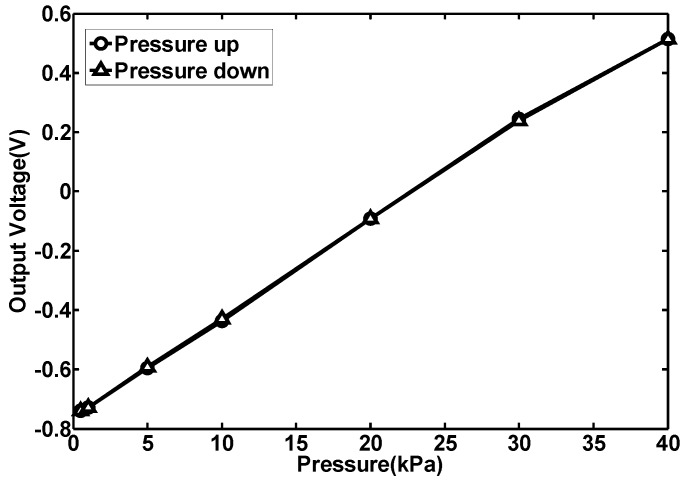
Hysteresis measurement results of the No. 1 structures.

The results show that the repeatability error is about 1.6% and the sensor shows excellent repeatability. To study the hysteresis of the sensor, the pressure is firstly increased from 0.5 kPa to 40 kPa and then decreased from 40 kPa to 0.5 kPa. The testing results are given in Figure 10, where the hysteresis error of the sensor is about 0.63% and the hysteresis characteristics of the sensor are quite good.

## 4. Conclusions

In this paper, an enhanced cross-beam piezoresistive pressure sensor has been developed. To indicate the improvement, two reference structures are designed. The sensor is fabricated by a process compatible with the CMOS process. The measurement results show that the sensitivity of this structure is significantly improved and compared with flat membrane structure, the improvement factor is about 3.8-fold. During measurement, it is also found that the sensor has some shortcomings. Further optimization will focus on how to improve the sensor sensitivity by choosing proper beam dimensions and put all four piezoresistors on the beam as well as get rid of the residual stress in the beam by choosing a suitable material or fabrication method. 
